# Determination of Transdermal Rate of Metallic Microneedle Array through an Impedance Measurements-Based Numerical Check Screening Algorithm

**DOI:** 10.3390/mi13050718

**Published:** 2022-04-30

**Authors:** Jingshan Mo, Junqing Liu, Shuang Huang, Baoming Liang, Xinshuo Huang, Cheng Yang, Meiwan Chen, Jing Liu, Tong Zhang, Xi Xie, Jun Guo, Fanmao Liu, Hui-Jiuan Chen

**Affiliations:** 1 School of Electronics and Information Technology, State Key Laboratory of Optoelectronic Materials and Technologies, Guangdong Province Key Laboratory of Display Material and Technology, Sun Yat-Sen University, Guangzhou 510006, China; mojsh5@mail2.sysu.edu.cn (J.M.); huangsh69@mail2.sysu.edu.cn (S.H.); liangbm@mail2.sysu.edu.cn (B.L.); huangxsh3@mail2.sysu.edu.cn (X.H.); yangch255@mail2.sysu.edu.cn (C.Y.); xiexi27@mail.sysu.edu.cn (X.X.); 2 Department of Cardiology, The First Affiliated Hospital of Jinan University, Guangzhou 510630, China; liujq0615@163.com; 3 State Key Laboratory of Quality Research in Chinese Medicine, Institute of Chinese Medical Sciences, University of Macau, Macau 999078, China; mwchen@um.edu.mo; 4 The First Affiliated Hospital of Sun Yat-Sen University, Guangzhou 510080, China; liuj753@mail.sysu.edu.cn; 5 School of Computer Science and Engineering, South China University of Technology, Guangzhou 510006, China; tony@scut.edu.cn; 6 Pazhou Lab, Guangzhou 510335, China

**Keywords:** microneedle, transdermal rate, COMSOL, impedance measurement

## Abstract

Microneedle systems have been widely used in health monitoring, painless drug delivery, and medical cosmetology. Although many studies on microneedle materials, structures, and applications have been conducted, the applications of microneedles often suffered from issues of inconsistent penetration rates due to the complication of skin-microneedle interface. In this study, we demonstrated a methodology of determination of transdermal rate of metallic microneedle array through impedance measurements-based numerical check screening algorithm. Metallic sheet microneedle array sensors with different sizes were fabricated to evaluate different transdermal rates. In vitro sensing of hydrogen peroxide confirmed the effect of transdermal rate on the sensing outcomes. An FEM simulation model of a microneedle array revealed the monotonous relation between the transdermal state and test current. Accordingly, two methods were primely derived to calculate the transdermal rate from the test current. First, an exact logic method provided the number of unpenetrated tips per sheet, but it required more rigorous testing results. Second, a fuzzy logic method provided an approximate transdermal rate on adjacent areas, being more applicable and robust to errors. Real-time transdermal rate estimation may be essential for improving the performance of microneedle systems, and this study provides various fundaments toward that goal.

## 1. Introduction

Point-of-care testing can facilitate health monitoring. By reducing the scale of laboratory equipment to achieve portability, advanced health monitoring can be performed, benefiting from advances in microfluidics, lab-on-a-chip technologies, and miniaturization [1,2]. As a result, point-of-care testing has evolved rapidly in recent years and is widely used for applications such as rapid detection of medical treatment [3,4,5,6], environmental factors [7,8], agricultural conditions [8], food characteristics [8], and quarantine aspects [9]. With the development of emergency medicine and intensive care medicine and the need for timely specimen detection, point-of-care testing has been recognized with wide utilization, from early urine glucose detection [6] to subsequent blood glucose detection [10], pregnancy tests [11], myocardial injury detection [12], and coagulation function evaluation [13,14,15], among others. Device miniaturization has enabled the development of wearable sensors for point-of-care testing, which will provide benefits through micro-invasive bio-mark detection and painless physiological monitoring, and also avoid time-consuming processes clinically.

Microneedles have been widely used in drug delivery [16,17] as well as other health [18,19] and cosmetology applications [20]. Attributing to inherent painless features, microneedles witnessed a significant material and structural evolution [21]. Microneedles can be categorized according to their solid or hollow geometry and dissolvability [22]. Materials and structures have intrinsic advantages and drawbacks, but early studies were conducted using only one material and one structure for a single purpose. Subsequently, composite structures were developed to outperform single structures. For example, coated microneedles combine the mechanical strength of state microneedles with the dose-controlled profile of dissolvable microneedles [23]. Recently, Li et al. demonstrated a closed-loop system for diabetes treatment using metallic microneedles for blood glucose detection and middle-hole microneedles for insulin delivery [10]. Among diverse microneedles, metallic sheet microneedles have excellent electrical conductivity and mechanical properties obtained from concise planar laser machining. Thus, they are the most widely commercialized microneedles and play a crucial role in advanced research, such as in vivo transdermal electroporation [24] and biomarker sensing [25].

Despite the obvious advantages of microneedle systems, several challenges remain to be addressed for accurate drug delivery and sensing. Remarkably, microneedles should achieve reliable skin penetration [19]. Considering the different characteristics of human or animal skin and the scattered and uneven stress caused by the arrangement of tips in microneedle arrays, piercing the skin may be complicated [26]. However, as most studies have focused on microneedle applications, the transdermal process has been mostly neglected. Some studies have been aimed at reducing the puncture force through geometric optimization based on finite element analysis of mechanics [26,27,28]. The needle tip width (radius) and wall angle were proved to be the crucial parameters affecting the puncture force [29,30]. Inspired by mosquitoes, Kim et al. found that skin pre-stretching and microneedle vibration may improve the insertion accuracy and reduce the insertion force [31]. In addition, bard microneedles have shown better retention in the skin [32,33,34]. Dyes are often used to characterize the transdermal status in microneedle studies. Specifically, the stained skin sections are used to observe the skin status after microneedle extraction to evaluate aspects such as the puncture depth, wound morphology, and drug diffusion range [23]. In addition, the stamp of the staining hole can be used to calculate the transdermal rate [35]. However, staining is a kind of posterior method which is unsuitable outside laboratory settings given concerns about the biosafety and economic efficiency of dyes. Moreover, the transdermal rate cannot be monitored in real-time using dyes, failing to guarantee the correct drug delivery dose. As the performance of microneedle sensors strongly depends on the electrode contact area, the transdermal rate should be monitored because it may affect the sensing results. In terms of drug delivery, an uncertain transdermal rate would lead to a larger dose deviation of the drug delivered through the microneedle array, affecting the drug delivery effect. Hence, accurate real-time transdermal rate estimation may improve the performance of microneedle systems.

**Figure 1:**
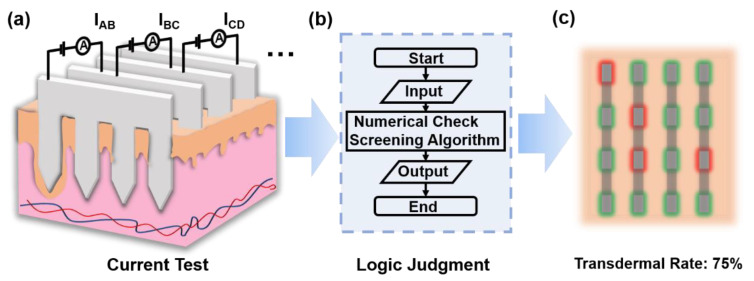
Schematic illustration of the impedance measurements-based numerical check screening algorithm. (**a**) The metallic microneedle arrays are inserted into the skin and the current between each row is measured respectively. (**b**) A logic judgment process is run with the measured current to calculate the transdermal rate. (**c**) The transdermal results are shown in two different colors. Red stands for the site where the microneedle is not penetrated, while green stands for the penetrated site by the microneedle.

In this study, we demonstrated a methodology for the determination of the transdermal rate of metallic microneedle array through impedance measurements-based numerical check screening algorithm (Figure 1). Three-electrode microneedle sensor composed of assembled metallic microneedle sheets fabricated by laser machining, where the microneedle sheets were functionalized with Pt by electrodeposition to allow sensing of hydrogen peroxide (H_2_O_2_) in solution. Microneedles with different number of tips were used to simulate different transdermal rates in experiments, where the in vitro sensing results, which indicated the transdermal rate, played an important role in sensing sensitivity, revealing the necessity for transdermal rate estimation. We applied a series of physics simulation models to study the relation between the transdermal rate and electrical variation at the microneedle–skin interface. The simulation results showed a negative correlation between the test current and the transdermal rate. Accordingly, we developed an impedance measurements-based numerical check screening algorithm for real-time transdermal rate estimation toward accurate biosensing using microneedle systems. The methodology demonstrated in this work provided a promising strategy for precise microneedles applications.

## 2. Materials and Methods

### 2.1. Fabrication of the Metallic Sheet Microneedle Sensor

**Figure 2:**
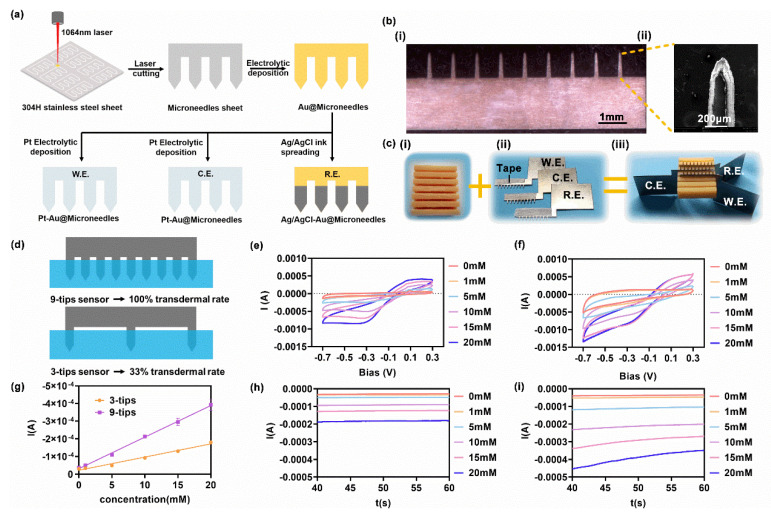
Fabrication of the metallic sheet microneedle array sensors and their sensing performance. (**a**) Fabrication of three−electrode metallic microneedle sheet. (**b**) (i) Optical micrograph of the microneedle sheet. (ii) SEM of the Pt/Au coated microneedle. (**c**) (i) Photograph of red resin frame case. (ii) Photograph of the metallic microneedle sheet with the tape sticking to the back. (iii) Photograph of the assembled sensor. (**d**) Schematic diagram of the H_2_O_2_ sensing experiment. (**e**,**f**) Scanning results of the three−tip sensor (**e**) and nine−tips sensor (**f**) in gradient H_2_O_2_ solution (0, 1, 5, 10, and 20 mM) by cyclic voltammetry (−0.7 to 0.3 V). (**g**) Steady−state amperometric responses (t > 40 s) were analyzed, and relations with H_2_O_2_ concentration were linearly fitted. (**h**,**i**) Respective amperometric responses of three-tip sensor (**h**) and nine-tip sensor (**i**) soaked in gradient H_2_O_2_ solutions. Bias = −0.3 V.

The metallic sheet microneedles were etched from 200 μm thick 304 H stainless steel plates using laser micro-etching. After etching, the microneedle sheets were laser cleansed and then soaked in a scaling powder solution (Yuexingda Electronic, Shenzhen, China) for 10 min. The microneedle sheets were deposited with a layer of gold for inner protection through electrodeposition (sulfite gold, 10 mA, 10 min). The working electrode sheets and counter electrode sheets were subsequently deposited with a layer of platinum using the same procedure (sulfite platinum, 10 mA, 10 min). Concurrently, referenced electrode sheets were obtained by applying Ag/AgCl ink (ALS, Tokyo, Japan) to the tips of the gold-coating microneedle sheets (Figure 2a). In addition, red resin frame cases were molded by 3D printing (Dongguan Broad Technology, Dongguan, China). After fabrication, a small piece of medical tape was attached to the back of the microneedle for mounting in the frame case. Reversible assembly was performed using rigid–elastomer–rigid mating (Figure 2c). The geometry of the microneedle and frame case is shown in Figure S1 of the Supporting Information.

### 2.2. In Vitro H_2_O_2_ Sensing

**Figure 3:**
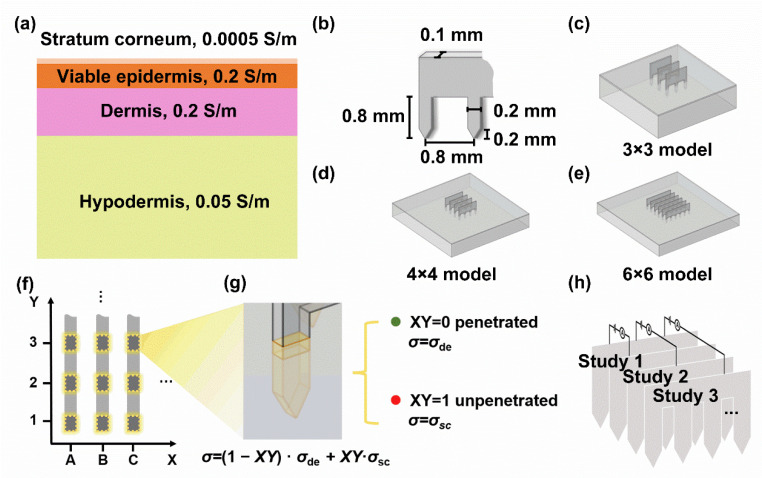
Schematic of the simulation model of metallic microneedle sheet array penetrating into the skin. (**a**) The anatomy of the skin and its electrical properties. The top to the bottom of the schematic represents the skin from the outside to the inside, involving stratum corneum, viable epidermis, dermis, and hypodermis, respectively. (**b**) Geometric sketch of the tip of the metallic microneedle sheet array. (**c**–**e**) Geometric sketch of 3 × 3, 4 × 4, and 6 × 6 metallic microneedle sheet array model. The distance between two microneedle sheet was 0.8 mm. (**f**) Diagram of the numbering of microneedle sheets and tips. The microneedle sheets were numbered by A, B, C, and so on. The tips on the microneedle sheet were numbered by 1, 2, 3, and so on. (**g**) Schematic of the tip–skin interface physic setting. The boundaries between tip and skin (highlighted by yellow) were set as contact impedance with 20 μm extra physical thickness. The conductivity of these boundaries was depended on the transdermal condition. (**h**) Schematic of the inter-sheet current study. The simulation results were obtained by the inter-sheet current study in pairs.

The electrochemical properties of the fabricated nine- and three-tip microneedle sensors were examined using a standard three-electrode electrochemical workstation (760E; CH Instruments, Austin, TX, USA). H_2_O_2_ concentrations of 0, 1, 5, 10, and 20 mM were added to a six-well plate (Corning, Corning, NY, USA). The sensor was placed vertically in the plate, and the tips were flooded with liquid (Figure 3b). At the intervals between tests, the tips were rinsed with deionized water and then dried with nitrogen blow. Both cyclic voltammetry and amperometry (I−t curves) were used to evaluate the electrochemical activity of the sensors. In addition, the sensitivity was obtained from linear regression of the amperometric response.

### 2.3. Finite Element Model

To explore the relation between transdermal rate and test current while the metallic microneedle array inserting to the skin, a series of finite element models were set up and studied by the electric current interface of COMSOL Multiphysics 5.5 (COMSOL, Burlington, MA, USA). As the simulation process included geometry, materials, physics, mesh, study, and results, details are shown below.

#### 2.3.1. Geometry and Materials

From the inside out, the skin is divided into hypodermis, dermis, viable epidermis, and stratum corneum. In general, the skin exhibits capacitive electrical properties, that is, providing a high-frequency electric excitation reduces the skin impedance [36]. Furthermore, the conductivity at each skin layer is different from its main component, mainly water, as illustrated in Figure 3a [37].

We established the models of 3 × 3, 4 × 4, and 6 × 6 microneedle arrays to explore the relation between transdermal rate and test current (Figure 3c–e). A microneedle sheet was 100 μm in thickness, and each microneedle was 200 μm in width and 800 μm in length, including the 200 μm tips. The tip spacing was the same as the sheet spacing of 800 μm (Figure 3b). The material of the microneedle sheet was iron (1 × 10^7^ S/m). Assuming the same conductivity for the viable epidermis and dermis, the skin model was simplified to two layers, with a 100 μm thick surface in the high-impedance layer (0.0005 S/m) and a 1.8 mm thick layer representing the conductive endothecium (0.2 S/m). The length of the skin block varied according to the model, ensuring that the microneedle array occupied one-ninth of the skin area in the center to weaken the fringe effect. The insertion depth of the microneedle was set to 600 μm.

#### 2.3.2. Physics Setup and Study Strategy

In COMSOL Multiphysics (COMSOL, Burlington, MA, USA), the circuit connecting two microneedle sheets was set as the default regarding geometry. A circuit was defined by setting one microneedle sheet as ground and another as voltage terminal. Thus, the skin impedance between two microneedle sheets was represented by the terminal current while applying a constant voltage (1 V in this study).

To determine the relation between transdermal rate and test current, the state (penetrated/unpenetrated) of each microneedle was recorded, obtaining 64, 256, and 4096 state combinations for the 3 × 3, 4 × 4, and 6 × 6 arrays, respectively. As building the geometry model for each combination was computationally expensive, physics settings and property parameterization were used for efficient simulation. First, each microneedle was denoted as *XY*, where *X* represents the sheet location and *y* represents the microneedle location in the sheet (Figure 3f). Then, the boundary between the inserted microneedle and skin was set as the contact impedance considering a 20 μm surface thickness (an extra thickness omitted in geometry). The electrical conductivity of each microneedle boundary was defined as:

(1−*XY*) ·*σ*_de_ + *XY*·*σ*_sc_,

where *σ*_de_ is the dermis conductivity and *σ*_sc_ is the stratum corneum conductivity. When *XY* is 1, its surface conductivity is equal to that of the stratum corneum, indicating an unpenetrated microneedle. When *XY* is 0, its surface conductivity is equal to that of the dermis, indicating a penetrated microneedle (Figure 3g). Thus, the microneedle state (penetrated/unpenetrated) was controlled by a pair of parameters (0 and 1), avoiding to rebuild the geometry of each transdermal condition.

An auxiliary parameter sweep in steady study was performed to combine all *XY* values. Therefore, all combinations of microneedle states were computed simultaneously (Figure 3h). Other physics boundary conditions and the mesh were set to their default values.

### 2.4. Estimation of Microneedle Array Characteristics

From the simulation results, we devised two methods for analysis: the exact method and the fuzzy logic method. The exact method allows to accurately determine the number of unpenetrated tips in each sheet but is rigorous regarding current testing. The fuzzy logic method only provides an approximate number of unpenetrated tips in an area, but it is more tolerant to deviations of the test current, thus being more applicable than the exact method. We performed the analyses using the LabVIEW graphical programming language (NI, Austin, TX, USA). The layout of the LabVIEW program for analysis is shown in Figures S2 and S3 of the Supporting Information.

## 3. Results and Discussion

### 3.1. In Vitro H_2_O_2_ Sensing

To verify the effect of the transdermal rate on sensing, we conducted an in vitro H_2_O_2_ sensing experiment. The nine- and three-tip sensors were evaluated with their tips immerged completely in the solutions, representing 100% and 33.3% transdermal rates, respectively (Figure 2d). H_2_O_2_, associated with tissue inflammation, is a biomarker of important mammalian physiological processes, and is an important reaction product of many enzyme-based biosensing methods.

Three electrode microneedle sheets were prepared by laser cutting of stainless-steel sheets, and treated by the subsequent processes including cleaning and electrodeposition of Au and Pt (Figure 1a). Such Pt-Au-Microneedle construction was proved to be stable, on account of the mature electrodeposition technology and the commercial electroplating solution [38].

The as-prepared blank microneedle sheet was characterized by the scanning electron microscope (SEM). The prepared microneedles were conformed to the design in geometric size, yet the laser-cut section is tilted attributed to the characteristics of laser processing itself, as the laser spot was not an absolute plane, but a Gaussian surface (Figure 1b). The medical tape served as the elastic cushion, while the three-electrode microneedle sheets were assembled into a 3D-printed red resin frame case to constitute the sensor (Figure 1c).

Cyclic voltammetry was first used to evaluate the electrochemical properties of the working electrode. The results of the three-tip and nine-tip sensors are shown in Figure 2e,f, respectively. Reduction peaks at −0.3 V were observed for both sensors, indicating successful H_2_O_2_ sensing. The current response was stronger in Figure 2e than in Figure 2f, indicating better sensing of the nine-tip sensor.

The amperometric response upon the increase of H_2_O_2_ concentration was also tested under −0.3 V bias. The steady-state currents (*t* > 40 s) of the three- and nine-tip sensors are shown in Figure 2h,i, respectively. Figure 2g shows the steady-state current and linear fitting results. The sensitivity of the nine-tip sensor was 17.88 μA/mM (*R^2^* = 0.9982), and that of the three-tip sensor was 7.47 μA/mM (*R^2^* = 0.9832). The two-fold difference in sensitivity indicated the number of microneedles in tissues could significantly affect the sensing performance, attributing to the different sensing area size.

### 3.2. Relation between Transdermal Rate and Test Current

To set up an impedance measurements-based numerical check screening algorithm for the determination of transdermal rate, the primary step was finding out the relation between transdermal rate and impedance in different transdermal conditions. Considering the vast transdermal conditions (4096 conditions for a pair of six-tip sheets) and the bias of the experiment, it was impractical and inaccurate to study such relation by experiment. In this work, a series of 3D microneedle–skin interaction finite element models were computed by the Electric Currents interface (ec) of COMSOL Multiphysics 5.5 (COMSOL, Burlington, MA, USA). Orderly, the microneedle sheets were labeled with capital letters (A, B, C, etc.), and the tips on the microneedle sheet were labeled with Arabic numerals (1, 2, 3, etc.). For example, “A2” referred to the second tip of the first sheet (Figure 2f). The transdermal condition (penetrated/unpenetrated) of each tip was parameterized (0/1) by the physics setting (Details were shown in the Materials and methods) (Figure 2g). Thus, the transdermal conditions could be exhaustively combined by the parametric sweep. The testing results of impedance were represented by test current, as the 1 V constant voltage source was served. The current between sheet A and sheet B was defined as “I_AB_”. Similarly, the testing results between two microneedle patches (e.g., sheet A and sheet C) were defined as “I_AC_”.

**Figure 4:**
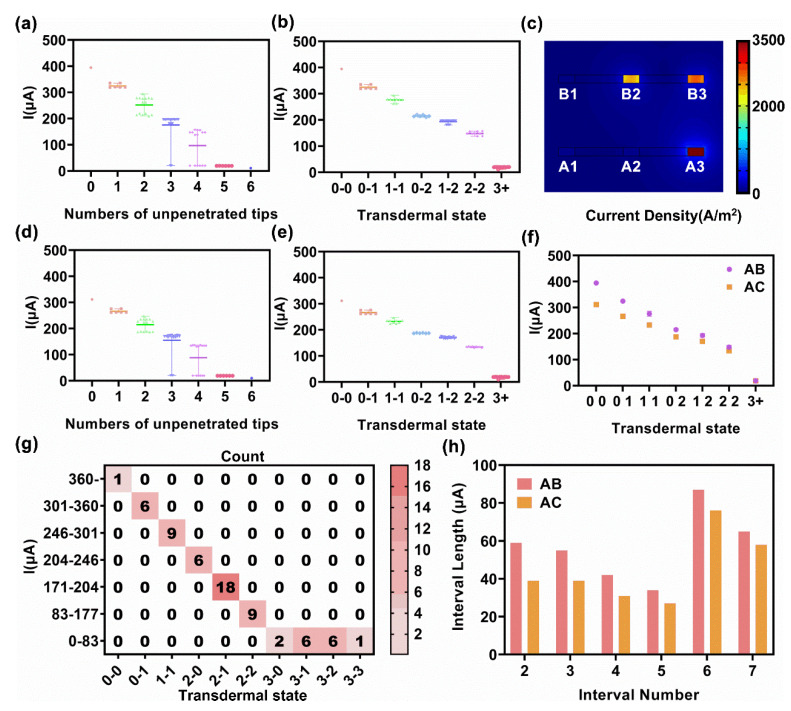
Simulation results of 3 × 3 microneedle array model, obtained from the statistical analysis of the terminal current after sweeping 64 transdermal events per pair of microneedle sheets. (**a**) The nested figure of the current distribution of A−B inter−sheet, divided by N_total_. (**b**) The nested figure of the current distribution of A−B inter-sheet, divided by the transdermal state. (**c**) A case diagram of the 2−1 transdermal state showing the nonuniform current contribution of the penetrated tips. The current density is represented by rainbow color. (**d**) The nested figure of the current distribution of A−C inter-sheet, divided by N_total_. (**e**) The nested figure of the current distribution of A−C inter-sheet, divided by the transdermal state. (**f**) The statistical results of the test current against the transdermal state. (**g**) Heat map of the current interval against the transdermal state of the A−B inter-sheet. The numerals in each cell referred to the count of transdermal events. (e.g., 18 events were enclosed into the “2−1 state”). (**h**) Histogram of the interval length of each interval. The x-axis started at interval 2 on account of that interval 1 was half open.

After sweeping all the combinations of transdermal conditions for the metallic sheet microneedle arrays, the simulation results were obtained for analysis, from simple to complex, starting with the 3 × 3 microneedle array model. At first, the 64 I_AB_ were divided into nests according to the total number of unpenetrated tips (N_total_). As shown in Figure 4a, the test current (y−axis) exhibited a monotonously negative correlation to the number of unpenetrated tips (x−axis) in general. While the x was greater than or equal to 3, the test currents were extremely low in some conditions. Because in these conditions, one of the two sheets was completely unpenetrated, equivalented to a large resistance in the test circuit. Moreover, while the x was equal to 2, the test currents were dispersed in the two groups, with mean values of 276 μA and 215 μA, respectively. Further analysis found that these two mean values belonged to two transdermal conditions. One was that both sheets had an unpenetrated tip, while the other case was where one microneedle sheet had two unpenetrated tips, and the tips of the other microneedle sheet were completely penetrated. Herein, a label including two numbers which were referred to the number of unpenetrated tips on the two tested sheets was defined as a transdermal state. Thus, the two above transdermal conditions could be simply described as “1−1 state” and “0−2 state”. Note that the two numbers were swappable, giving a “0−2 state” from I_AB_; there could be two unpenetrated tips in microneedle sheet A or microneedle sheet B. The “2−0 state” was defaulted as it referred to the same transdermal condition of the “0−2 state”. Thus, the precise number of the unpenetrated tips of the two tested sheets could not be obtained according to a single test current.

A more detailed division according to transdermal states was shown in Figure 4b. The test current exhibited a one−to−one corresponding and monotonously negative correlation to the transdermal state. The currents that responded to the same transdermal state were centralized but were not in full accord, fluctuating around the mean value. The variations were caused by the position difference of the penetrated tips. As the essence of the current test was that the resistance between two microneedles sheets was different from different transdermal conditions, the relative position of the tips would impact their current contribution. For example, Figure 4c showed the current density of a condition of 2-1 transdermal state which tips A1, A2, and B1 were unpenetrated. The current densities in the penetrated tips were different, the current density in B3 was bigger than B2 because B3 was closer to A3, and the current density in A3 was undisputedly biggest because it equated to the sum of B2 and B3. The 3+ state was a special transdermal state, including the 3−0, 3−1, 3−2, and 3−3 state, and was termed the non-conductive transdermal state. In the 3+ state, one of the two sheets was completely unpenetrated; hence, a negligible conductive pathway resulted in a low current. In the non-conductive transdermal state, the one-to-one correspondence between the transdermal state and test current was lost because the value of a resistor cannot be measured when connected in series with an extremely large resistance (i.e., open circuit).

Figure 4d,e demonstrates the current test results between sheet A and sheet C. The nest distribution of I_AC_ was almost identical to I_AB_, except that the overall current range was narrower. As the distance between sheet A and C was 1600 μm and that of sheet A and B was 800 μm, the increased distance led to the weakened current response. The mean values of each transdermal state of the 3 × 3 microneedle array model were shown in Figure 4f, and the current was divided into intervals according to these mean values, for subsequently estimating the number of unpenetrated tips by test current. The boundary of each interval was the mean value of the mean value of the adjacent state. The details of the division of the 3 × 3 microneedle array model are provided in Table 1.

The division results of I_AB_ were obtained as heat maps, where the number in the cells indicated the count of transdermal substrates. A transdermal substate represented the transdermal state but with different unpenetrated tip positions (Figure 4g). I_AC_ showed the same distribution of cells in heat maps, but with a narrow interval current length (Figure S4). The interval length was compared in the histogram (Figure 4h). The length of interval 1 was negligible because it was a half-open interval. In general, the interval length of I_AC_ is smaller than that of I_AB_. The interval length was diminished gradually from interval 2 to 5. As for intervals 6 and 7, the low current of the 3+ state provided a wide current range between the 2-2 state and the 3+ state, leading to the two long length intervals.

**Figure 5:**
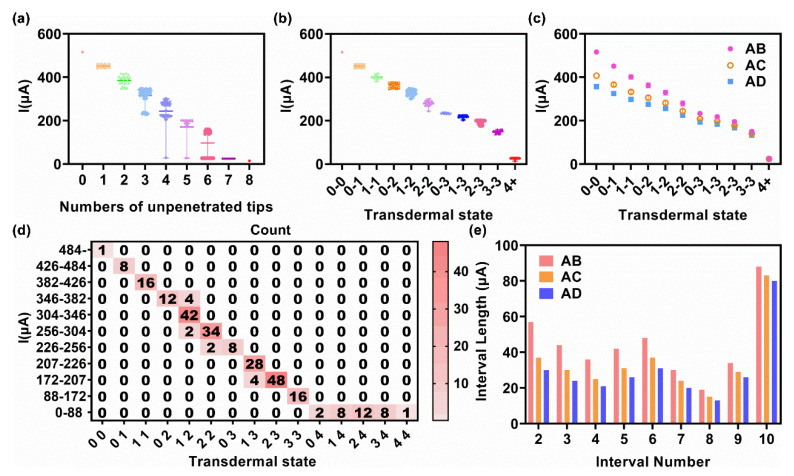
Simulation results of 4 × 4 microneedle array model, obtained from the statistical analysis of the terminal current after sweeping 256 transdermal events per pair of microneedle sheets. (**a**) The nested figure of the current distribution of A–B inter–sheet, divided by N_total_. (**b**) The nested figure of the current distribution of A–B inter–sheet, divided by transdermal state. (**c**) The statistical results of the test current against the transdermal state. (**d**) Heat map of the current interval against the transdermal state of A–B. Simulation results of 3 × 3 microneedle array model, obtained from the statistical analysis of the terminal current after sweeping 64 transdermal events per pair of microneedle sheets. The numerals in each cell referred to the count of transdermal events (e.g., 16 events were enclosed into the “1–1 state”). (**e**) Histogram of the interval length of each interval. The x–axis started at interval 2 on account that interval 1 was half–open.

The results of the 4 × 4 microneedle array model were demonstrated in Figure 5 and the interval division was shown in Table 2. Compared with the results of the 3 × 3 microneedle array model, the results of the 4 × 4 microneedle array model were a little more chaotic while dividing them via the total number of unpenetrated tips. In the results of the A-B inter-sheet of the 3 × 3 microneedle array model, the current and the total number of unpenetrated tips maintained a monotonously negative correlation, excepting the test currents of non-conductive states, which were sinking in the bottom of the nested figure as low current values (Figure 4a). However, the current ranges overlap, while the total number of unpenetrated tips were equaled to 3 and 4 in I_AB_ of the 4 × 4 model (Figure 5a). Yet, the current was still monotonously negative related to the transdermal state (Figure 5b). According to the rank of the transdermal state, the primary factor in the test current was the maximum number of unpenetrated tips in a single sheet, instead of the total number of unpenetrated tips of two sheets.

The heat map of I_AB_ for the 4 × 4 microneedle array model is shown in Figure 5d. The one-to-one correspondence between the current and conductive transdermal state was slightly skewed, as two transdermal states occurred in some intervals. To explain this phenomenon, the current state and state in interval 4 were exhaustively considered in a scatter diagram (Figure 5b). Some test currents (346.47–346.69 μA) of the 1-2 state were much higher than the mean current (329 μA) and very close to some test currents (348.09–348.11 μA) of the 0-2 state. Thus, a test current in interval 4 had a 25% probability with respect to the 1-2 state. Although the boundary can be set to 347 μA to obtain a one-to-one correspondence, the correspondence was highly sensitive to a test current error. Nevertheless, states 0-2 and 1-2 had almost the same number of unpenetrated tips, and the correspondence was still considerable. Similar results were obtained for the I_AC_ and I_AD_ of 4 × 4 microneedle array models, specifically not repeating them.

**Figure 6:**
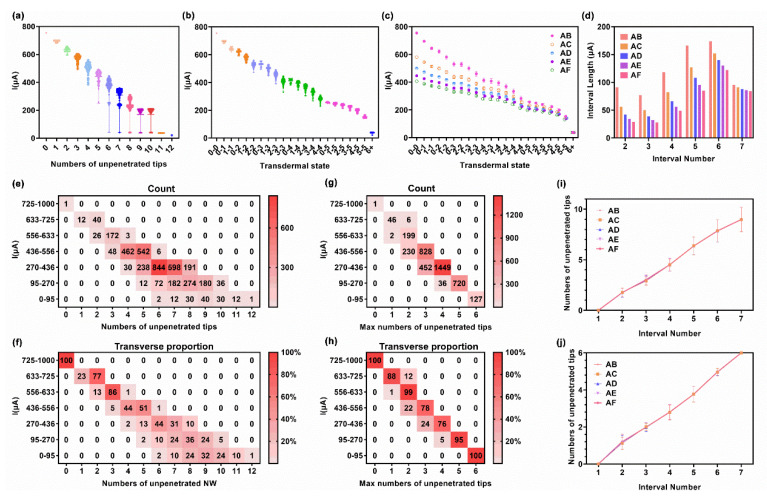
Simulation results of the 6 × 6 microneedle array model, obtained from the statistical analysis of the terminal current after sweeping 4096 transdermal events per pair of microneedle sheets. (**a**) The nested figure of the current distribution of A-B inter-sheet, divided by N_total_. (**b**) The nested figure of the current distribution of A–B inter-sheet, divided by the transdermal state. (**c**) The statistical results of the test current against the transdermal state. (**d**) Histogram of the interval length of each interval. The x–axis started at interval 2 on account of that the interval 1 was half open. (**e**) The counting heat map of the current interval against the N_total_ of A–B inter–sheet. (**f**) The percentage heat map of the current interval against the N_total_ of A–B inter-sheet. The numerals in each cell referred to the probability of N_total_ in certain current intervals. (**g**) The counting heat map of the current interval against the N_max__–__s_ in a single sheet of A-B inter-sheet. (**h**) The percentage heat map of the current interval against the N_max__–__s_ of A-B inter–sheet. The numerals in each cell referred to the probability of N_max__–__s_ in certain current intervals. (**i**) The statistical results of the N_total_ of each interval in the A–B inter–sheet. (**j**) The statistical results of N_max__–__s_ in a single sheet of each interval in the A–B inter–sheet.

As more tips were placed on a sheet, the chaos level increased. In I_AB_ of the 6 × 6 microneedle array model, the current range overlap was not only occurred in the results divided by the total number of unpenetrated tips (Figure 6a), but also in the results divided by transdermal states (Figure 6b). The mean value of the test current and transdermal state preserved a negative correlation, but the distance between the mean values of adjacent states was small (Figure 6c). Thus, it was difficult to divide the interval having a one-to-one correspondence into a transdermal state. Hence, several states were grouped for a given current for the group and the test current to show a one-to-one correspondence (Table 3). Figure 6e shows the current interval according to the total number of unpenetrated tips. Several transdermal conditions with different numbers of unpenetrated tips appeared in one current interval. As the test current value decreased, more transdermal conditions were mapping to one current interval, where there was only one transdermal condition (N_total_ = 0) in the first current interval (I = 725–1000 μA), there were seven transdermal conditions (N_total_ = 6, 7, 8, 9, 10, 11, and 12) in the last current interval (I = 0–95 μA). The numerals in the cell of the graph referred to the count of transdermal events (i.e., transdermal substate), with an approximately normal distribution emerging. Lengthways, interval 5 (I = 270–436 μA) contained the most events, followed by interval 4 (I = 436–556 μA) and 6 (I = 95–270 μA), and the remaining interval accounted for only 9.3% of the total events. Breadthways, in each interval, the events were concentrated in the transdermal condition in which the number of unpenetrated tips was medium, obtaining the transverse probability shown in Figure 6f. According to the probability of each transdermal condition, the mean and deviation of the number of unpenetrated tips per interval were calculated (Figure 6i). This result can be used to roughly determine the transdermal rate based on the test current.

Figure 6g showed the current interval according to the maximum number of unpenetrated tips in a single sheet (N_max-s_) for a transdermal state. The number of transdermal conditions in each interval substantially decreased in this relation. There were not more than two transdermal conditions in a current interval, and the probability of most likely transdermal condition was more than 76% (Figure 6h). Figure 6j shows the mean and deviation of the N_max-s_ per interval, calculating from each N_max-s_ and its probability. The deviation substantially decreased compared with that shown in Figure 6i. However, this method only provided the largest number of unpenetrated tips in a pair of sheets, while the other side was unknown.

According to the above discussion, the following conclusions are summarized: (1) The test current exhibited a negative correlation to the transdermal state; (2) The increased tips in a sheet would break the one-to-one correspondence and monotony of the above correlation; (3) The current response would weaken as the distance between two sheets increased. Thus, the test current between adjacent sheets was more reliable for estimation, whereas the test current between two sheets over a large span was unreliable.

### 3.3. Transdermal Rate Evaluation via a Test Current

**Figure 7:**
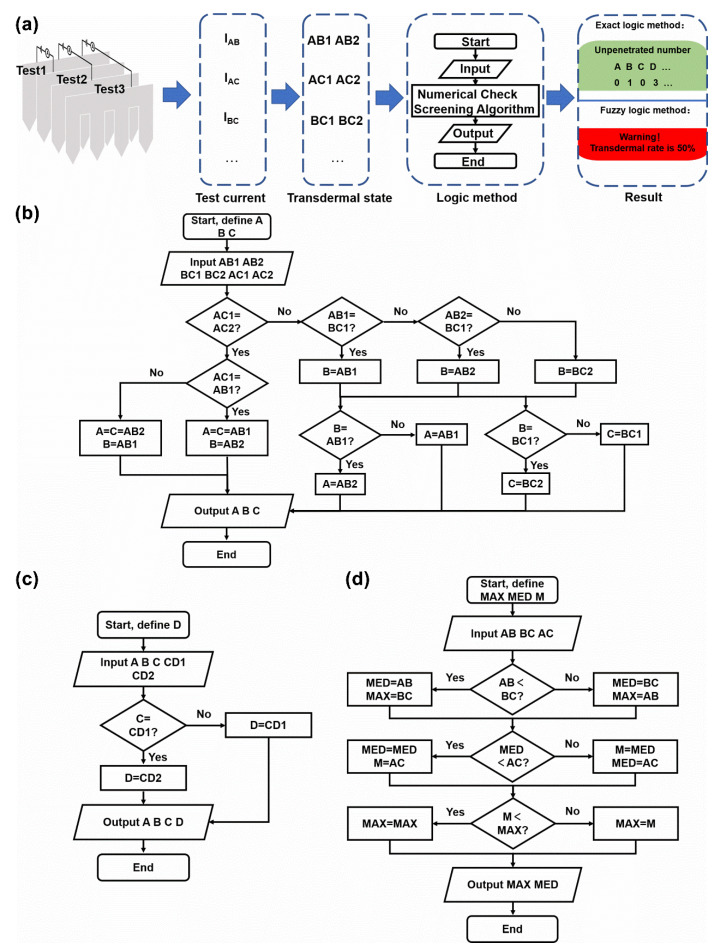
The logical principle for transdermal rate evaluation via the test current. (**a**) The workflow of the designed logic judgment system. (**b**) The logic diagram of the exact logic method for the three sheets area. A, B, and C were the unknown variates referring to the N_exact_ of the microneedle sheet A, B, and C. AB1 and AB2 were the input referring to the transdermal state against I_AB_. Similar definition to BC1, BC2, AC1, and AC2. (**c**) The logic diagram of the exact logic method for the adding sheet. This work branch was based on the result of Figure 7b. D is the unknow variate referring to the N_exact_ of the microneedle sheet D. CD1 and CD2 were the input referring to the transdermal state against I_CD_. (**d**) The logic diagram of the fuzzy logic method for the three sheets area. MAX and MED are the unknow variates referring to N_MAX_ and N_MED_, and Variate M is the temporary storage. AB, CD, and EF are the input referring to the N_max-s_ for the corresponding sheet pairs.

The relation between the test current and transdermal state of a pair of sheets was explored. To determine the transdermal rate of the microneedle array using the test current, the next step was constructing a numerical check screening algorithm utilizing the inner relation between these transdermal states. The workflow of the designed system is shown in Figure 7a. For a working microneedle array sensor, the inter-sheet current (I_AB_, I_AC_, I_BC_, etc.) was tested in pairs, mapping certain transdermal states. Then, the transdermal states were taken as the input of the logic processing module, and the transdermal conditions of the microneedle array were obtained through logical calculation.

#### 3.3.1. Exact Method

From the simulation results, one test current corresponded to one transdermal state, including two numbers of unpenetrated tips for two tested sheets, but these numbers were uncertain for the exact sheet. The proposed exact method depicted in Figure 7b was used to determine the number of unpenetrated tips per sheet. Starting with the 3 × 3 microneedle array model, variants A, B, and C were defined to represent the exact number of unpenetrated tips (N_exact_) in relevant sheets. Herein, the two numbers of the transdermal state mapping to I_XX_ were marked as “XX1” and “XX2”. The footnote “XX” refers to a pair of sheets in one current test. For the three test currents of the three sheets, six numbers of unpenetrated tips (AB1, AB2, BC1, BC2, AC1, and AC2) were determined and inputted into the logic module. First, the equality between AC1 and AC2 was evaluated. While AC1 was equal to AC2, meaning that A was equal to C, the method only needed to determine whether AB1 or AB2 was equal to B. While AC1 was equal to AB1, AB2 was equal to B and vice versa. Hence, A, B, and C were found out in this branch and output. In another branch, while AC1 was not equal to AC2, two equal numbers among AB1, AB2, BC1, and BC2 were required to be determined and assigned to B. As these four numbers represented unpenetrated tips of three microneedle sheets, two of them were definitely equal to the number of unpenetrated tips for the middle microneedle sheet B. Specifically, the equality of BC1 to AB1 or AB2 was determined and assigned to B. If neither AB1 nor AB2 was equal to BC1, BC2 was assigned to B. Next, comparing AB1 and AB2 with B, the unequal value was assigned to A. Finally, comparing BC1 and BC2 with B, the unequal value was assigned to C (Figure 7b). Thus, the exact number of unpenetrated tips for the 3 × 3 array was determined through the test current and logic derivation. For the 4 × 4 microneedle array, an additional flow was used to determine the number of unpenetrated tips of sheet D, based on the procedure for the 3 × 3 microneedle array. Variants D was defined to represent the number of unpenetrated tips in the microneedle sheet D. CD1 and CD2 were the two numbers of the transdermal state mapping to I_CD._ Comparing C and CD1, if C was equal to CD1, CD2 was assigned to D. Otherwise, CD1 was assigned to D (Figure 7c).

Ideally, the number of unpenetrated tips for an n × n microneedle array can be exactly determined by adding more procedure flows successively. After determining the N_exact_ of the microneedle sheet A, B, and C, the N_exact_ of the microneedle sheet D can be determined based on the N_exact_ of the microneedle sheet C. Analogously, the N_exact_ of the microneedle sheet E can be determined based on the N_exact_ of the microneedle sheet D, and so on. However, as more tips are added in a microneedle sheet, the one-to-one correspondence between the test current and transdermal state was lost (Figure 5b). Thus, the above exact logic method is only applicable for microneedles arrays with a small number of tips (e.g., 3 × 3 or 4 × 4 microneedle arrays), but inapplicable for massive microneedle arrays (e.g., 6 × 6 microneedle arrays).

#### 3.3.2. Fuzzy Logic Method

The mapping in Table 3 was structured according to the simulation results. One test current interval was correlated to several pairs of numbers of unpenetrated tips with the same N_max-s_. Thus, the simplest method for transdermal rate estimate was summing 1.5 times the maximum of each two sheets. For the 6 × 6 microneedle array, the following formulation can be applied:

N = 1.5 × (AB + CD + EF)

where N is the number of unpenetrated tips, and AB, CD, and EF were the maximum numbers of unpenetrated tips for the corresponding sheet pairs. Factor 0.5 was a guess value for the unknown number of unpenetrated tips. There was a 1/2 rate of uncertain values in the abovementioned method, which could be reduced by applying fuzzy logic.

As a test current maps to the maximum number of unpenetrated tips of a pair of sheets, the minimum number of the unpenetrated tips of a pair of sheets was unknown.

Considering three microneedle sheets (A, B, and C), there were three actual number of the unpenetrated tips of each sheet. After the current test in pairs, I_AB_, I_BC_, and I_AC_ could reveal the N_max-s_ of A-B inter-sheet, B-C inter-sheet, and A-C inter-sheet, yet the smallest value of the actual number of the unpenetrated tips of microneedle sheet A, B, and C was still unknown. Thus, three tests between three sheets confirmed two actual numbers of unpenetrated tips, the maximum and median. The maximum and median could be determined by comparing the three test results in pairs as shown in Figure 7d. Variates MAX and MED were defined referring to the N_MAX_ and N_MED_, and Variate M was defined for temporary storage. AB, BC, and AC were the input referring to the maximum numbers of unpenetrated tips for the corresponding sheet pairs. Firstly, AB and BC were compared; the large one was assigned to MAX and the small one was assigned to MED. Next, MED and AC were compared; the large one was assigned to M and the small one was assigned to MED. Finally, M and MAX were compared; the large one was assigned to MAX. Thus, the maximum and median were found.

In this way, the uncertainty rate reduced from 1/2 to 1/3. The formulation for the three microneedle sheets can be expressed as:

N = N_MAX_ + N_MED_ + 0.5N_MED_

where N_MAX_ and N_MED_ are the maximum and the median number of unpenetrated tips in the sheets, respectively. Factor 0.5 for the median was a guess for the minimum.

## 4. Conclusions

We fabricated a metallic sheet microneedle sensor using laser machining and 3D printing. The electrochemical properties of three- and nine-tip sensors were evaluated by cyclic voltammetry and amperometry, revealing that the transdermal rate considerably impacts sensing. Then, simulation models were built to study the relation between the transdermal rate and test current. By defining the transdermal state, its linear relation with the test current was determined. However, the one-to-one correspondence was gradually lost with more microneedle tips in the array. Then, two methods were devised to determine the transdermal rate from the test current. The exact method accurately provided the number of unpenetrated tips on each sheet based on the one-to-one correspondence between the transdermal state and test current. The fuzzy logic method provided the approximate transdermal rate on an adjacent area with robustness against test inaccuracies. These methods provide transdermal rate estimation of metallic microneedle arrays toward real-time estimation.

## Figures and Tables

**Table 1:** The current interval division of the transdermal state of the 3 × 3 microneedle array model.

3 × 3 Array		I_AB_		I_AC_	
Interval Number	Transdermal State	Current Range (μA)	Interval Length (μA)	Current Range (μA)	Interval Length (μA)
1	0-0	360-	-	289-	-
2	0-1	301–360	59	250–289	39
3	1-1	246–301	55	210–250	39
4	0-2	204–246	42	179–210	31
5	1-2	171–204	34	152–179	27
6	2-2	83–177	87	76–152	76
7	3+	0–83	65	0–76	58

**Table 2:** The current interval division of the transdermal state of the 4 × 4 microneedle array model.

4 × 4 Array		I_AB_		I_AC_		I_AD_	
Interval Number	Transdermal State	Current Range (μA)	Interval Length (μA)	Current Range (μA)	Interval Length (μA)	Current Range (μA)	Interval Length (μA)
1	0-0	484-	-	387-	-	341-	-
2	0-1	426–484	57	349–387	37	311–341	30
3	1-1	382–426	44	319–349	30	287–311	24
4	0-2	346–382	36	294–319	25	266–287	21
5	1-2	304–346	42	263–394	31	240–266	26
6	2-2	256–304	48	226–263	37	209–240	31
7	0-3	226–256	30	203–226	24	189–209	20
8	1-3	207–226	19	187–203	15	176–189	13
9	2-3	172–207	34	159–187	29	150–176	26
10	3-3	88–172	84	83–159	76	80–150	70
11	4+	0–88	88	0–83	83	0–80	80

**Table 3:** The current interval division of the transdermal state of the 6 × 6 microneedle array model.

6 × 6 Array		I_AB_		I_AC_		I_AD_		I_AE_		I_AF_	
Interval Number	Transdermal State	Current Range (μA)	Interval Length (μA)	Current Range (μA)	Interval Length (μA)	Current Range (μA)	Interval Length (μA)	Current Range (μA)	Interval Length (μA)	Current Range (μA)	Interval Length (μA)
1	0-0	725-	-	562-	-	486-	-	436-	-	397-	-
2	0-1, 1-1	633–725	91	506–562	56	443–486	42	401–436	34	368–397	29
3	0-2, 1-2	556–633	77	455–506	50	403–443	39	369–401	32	340–368	28
4	0-3, 2-2, 1-3, 2-3	436–556	118	373–455	82	337–403	66	312–369	56	291–340	49
5	0-4, 3-3, 1-4, 2-4, 3-4, 4-4	270–436	166	245–373	127	229–337	108	217–312	95	206–291	85
6	0-5, 1-5, 2-5, 3-5, 4-5, 5-5	95–270	174	92–245	152	89–229	140	86–217	130	84–206	122
7	6+	0–95	95	0–92	91	0–89	88	0–86	86	0–84	84

## Data Availability

In this section, please provide details regarding where data supporting reported results can be found, including links to publicly archived datasets analyzed or generated during the study. Please refer to suggested Data Availability Statements in section “MDPI Research Data Policies” at https://www.mdpi.com/ethics (accessed on 28 April 2022). You might choose to exclude this statement if the study did not report any data.

## Supplementary Materials

The following supporting information can be downloaded at: https://www.mdpi.com/article/10.3390/mi13050718/s1. Figure S1: (a) The CAD design drawing of the metal microneedles sheet. (i) The CAD design drawing of a nine-tip sheet. (ii) The CAD design drawing of a three-tip sheet. (iii) The enlarged drawing of the tip. (b) The CAD design drawing of the resin frame case. (i) The main view of the resin frame case drawing. (ii) The left view of the resin frame case drawing; Figure S2: The LabVIEW layout design of the exact logic method. (a) The “true” branch which AC1 is equal to AC2. (b) The “false” branch in which AC1 is unequal to AC2. (i) The input control, inputting the numbers of the transdermal state as the starting point. (ii) Comparison with the control to detect whether the two inputs are equal or not. The output of this control is the Boolean value. (iii) The select control; while the Bool input was 1, the output number was inputted in the true channel, and vice versa. (iv) The case structure, while the Bool input was 1, the “true” branch was executed, and vice versa. (v) The local variable refers to the (vi) output variable; Figure S3: The LabVIEW layout design of the fuzzy logic method. (i) The input control, inputting the maximum numbers of the unpenetrated tips of the two sheets as the starting point. (ii) The maximum and minimum control, after comparing the two input values, output the large one in the top channel and the small one in the bottom channel. (iii) The output variable of the logic flow; Figure S4: Heat map of the current interval of the transdermal state of A-C of the 3 × 3 model; Figure S5: Heat map of the current interval of the transdermal state of (a) A-C and (b) A-D of the 4 × 4 model; Figure S6: The counting heat map of the current interval of the Nmax-s in a single sheet of (a) A-C, (c) A-D, (e) A-E, and (g) A-F of the 6 × 6 model. The percentage heat map of the current interval of the Nmax-s in a single sheet of (b) A-C, (d) A-D, (f) A-E, and (h) A-F of the 6×6 model; Figure S7: The counting heat map of the current interval of the Ntotal of (a) A-C, (c) A-D, (e) A-E, and (g) A-F of the 6 × 6 model. The percentage heat map of the current interval of the Ntotal of (b) A-C, (d) A-D, (f) A-E, and (h) A-F of the 6 × 6 model.
