# Supplementary material for: Determination of Transdermal Rate of Metallic Microneedle Array through an Impedance Measurements-Based Numerical Check Screening Algorithm
Source: Micromachines (Basel). 2022 Apr 30;13(5):718. doi: 10.3390/mi13050718 (PMC9146767; doi:10.3390/mi13050718)
Supplement: Supplementary file 1 [file micromachines-13-00718-s001.zip › micromachines-1675963-supplementary.pdf]

micromachines

Article

Determination of Transdermal Rate of Metallic Microneedle Array through an Impedance Measurements-Based Numerical Check Screening Algorithm

Jingshan Mo [1], Junqing Liu [2], Shuang Huang [1], Baoming Liang [1], Xinshuo Huang [1], Cheng Yang [1], Meiwan Chen [3], Jing Liu [4], Tong Zhang [5,6], Xi Xie [1,4], Jun Guo [2,*], Fanmao Liu [4,*] and Hui-Jiuan Chen [1,*]

1 School of Electronics and Information Technology, State Key Laboratory of Optoelectronic Materials and Technologies, Guangdong Province Key Laboratory of Display Material and Technology, Sun Yat-Sen University, Guangzhou 510006, China; mojsh5@mail2.sysu.edu.cn (J.M.); huangsh69@mail2.sysu.edu.cn (S.H.); liangbm@mail2.sysu.edu.cn (B.L.); huangxsh3@mail2.sysu.edu.cn (X.H.); yangch255@mail2.sysu.edu.cn (C.Y.); xiexi27@mail.sysu.edu.cn (X.X.)
2 Department of Cardiology, the First Affiliated Hospital of Jinan University, Guangzhou 510630, China; liujq0615@163.com
3 State Key Laboratory of Quality Research in Chinese Medicine, Institute of Chinese Medical Sciences, University of Macau, Macau 999078, China; mwchen@um.edu.mo
4 The First Affiliated Hospital of Sun Yat-Sen University, Guangzhou 510080, China; liuj753@mail.sysu.edu.cn
5 School of Computer Science and Engineering, South China University of Technology, Guangzhou 510006, China; tony@scut.edu.cn
6 Pazhou Lab, Guangzhou 510335, China
* Correspondence: dr.guojun@163.com (J.G.); liufm9@mail.sysu.edu.cn (F.L.); chenhuix5@mail.sysu.edu.cn (H.-J.C.)

Citation: Mo, J.; Liu, J.; Huang, S.; Liang, B.; Huang, X.; Yang, C.; Chen, M.; Liu, J.; Zhang, T.; Xie, X.; et al. Determination of Transdermal Rate of Metallic Microneedle Array through an Impedance Measurements-Based Numerical Check Screening Algorithm. *Micromachines* **2022**, *13*, 718. https://doi.org/10.3390/mi13050718

Academic Editors: Seong-O Choi and Nam-Trung Nguyen

Received: 26 March 2022
Accepted: 28 April 2022
Published: 30 April 2022

Publisher's Note: MDPI stays neutral with regard to jurisdictional claims in published maps and institutional affiliations.

Copyright: © 2022 by the authors. Licensee MDPI, Basel, Switzerland. This article is an open access article distributed under the terms and conditions of the Creative Commons Attribution (CC BY) license (http://creativecommons.org/licenses/by/4.0/).

Supplementary information

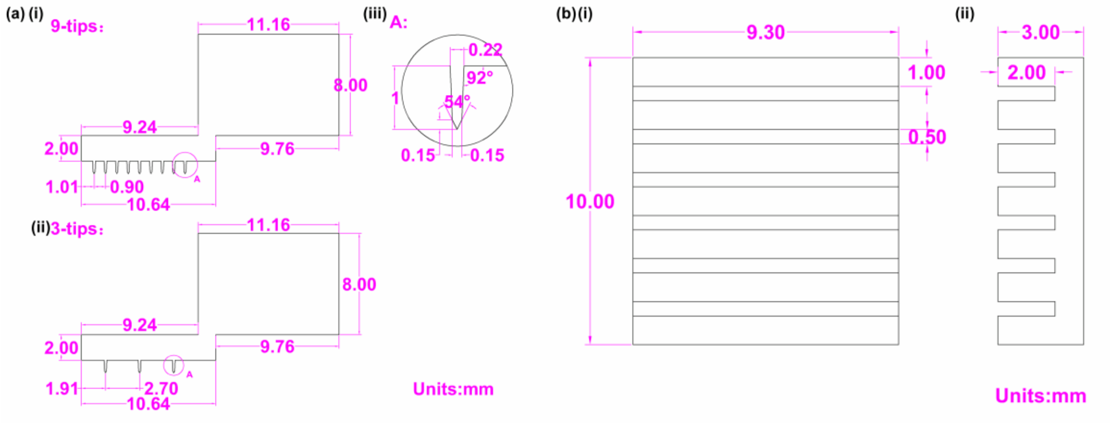

Figure S1. (a)The CAD design drawing of metal microneedles sheet. (i)The CAD design drawing of a 9-tips sheet. (ii)The CAD design drawing of a 3-tips sheet. (iii)The enlarged drawing of the tip. (b) The CAD design drawing of resin frame case. (i) The main view of resin frame case drawing. (ii) The left view of resin frame case drawing.

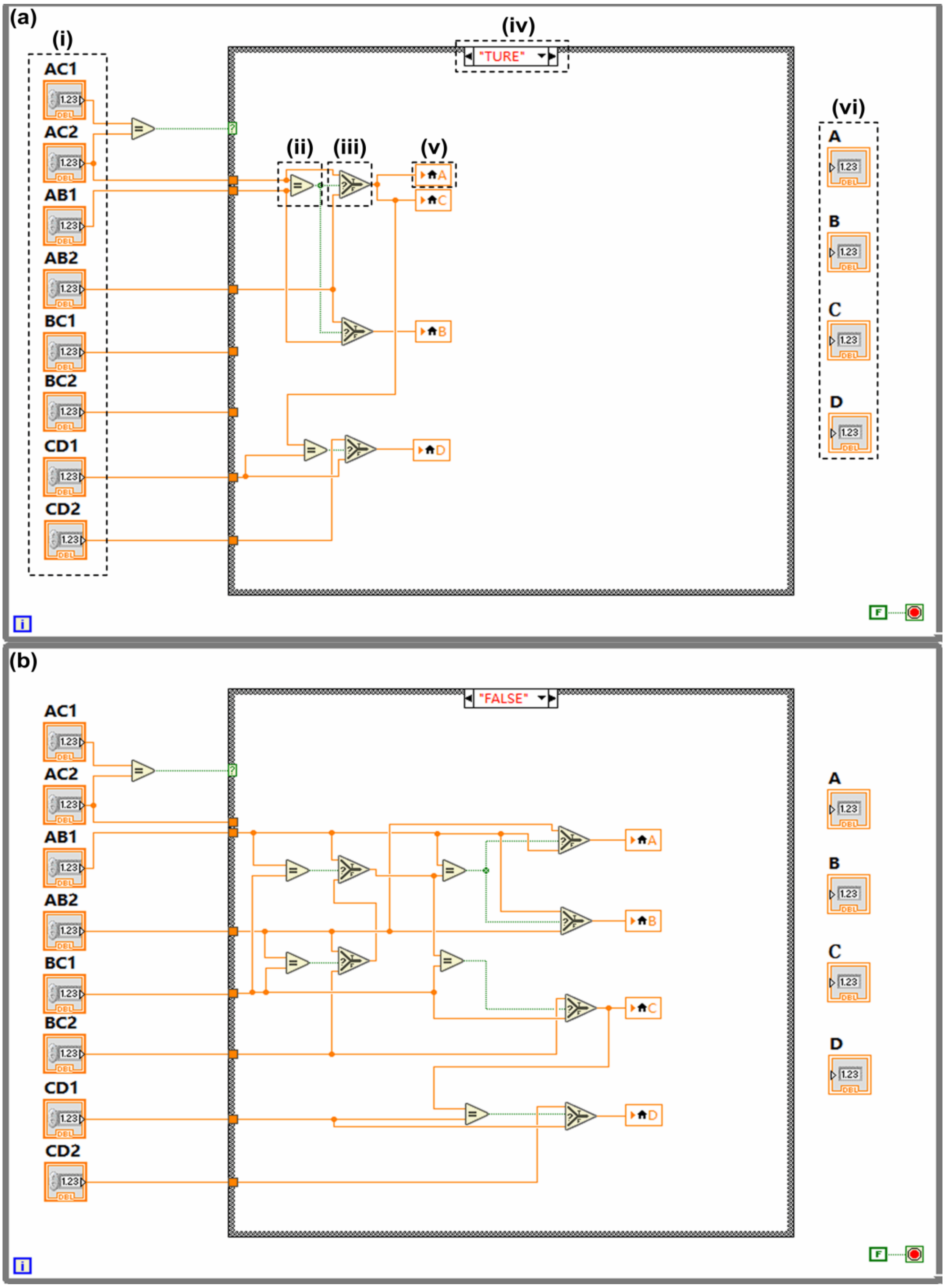

Figure S2. The LabVIEW layout design of the exact logic method. (a) The "true" branch which AC1 is equal to AC2. (b) The "false" branch in which AC1 is unequal to AC2. (i)The input control, input the numbers of the transdermal state as the starting point. (ii) The compare control to detect whether the two inputs are equal or not. The output of this control is the Boolean value. (iii) The select control, while the Bool input was 1, output the number inputted in the true channel, vice versa. (iv) The case structure, while the Bool input was 1, execute the "true" branch, vice versa. (v)The local variable refers to the (vi) output variable.

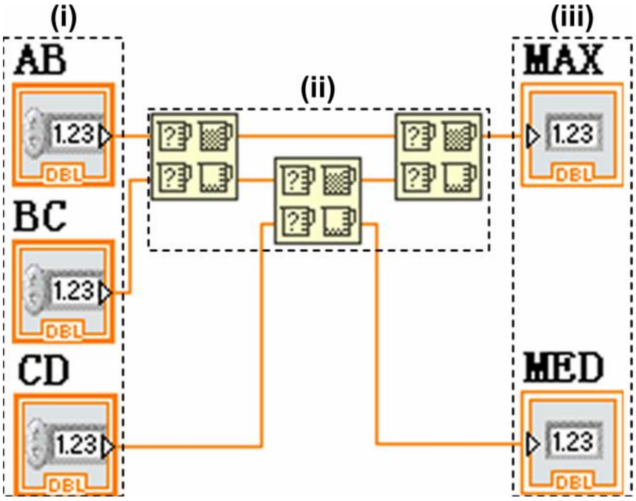

Figure S3. The LabVIEW layout design of the fuzzy logic method. (i) The input control, inputting the maximum numbers of unpenetrated tips of the two sheets as the starting point. (ii) The maximum and minimum control, after comparing the two input values, output the large one in the top channel, the small one in the bottom channel. (iii) The output variable of the logic flow.

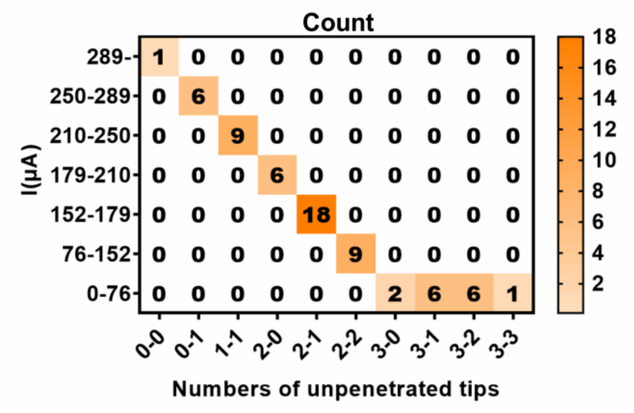

Figure S4. Heat map of the current interval of the transdermal state of A-C of the 3×3 model.

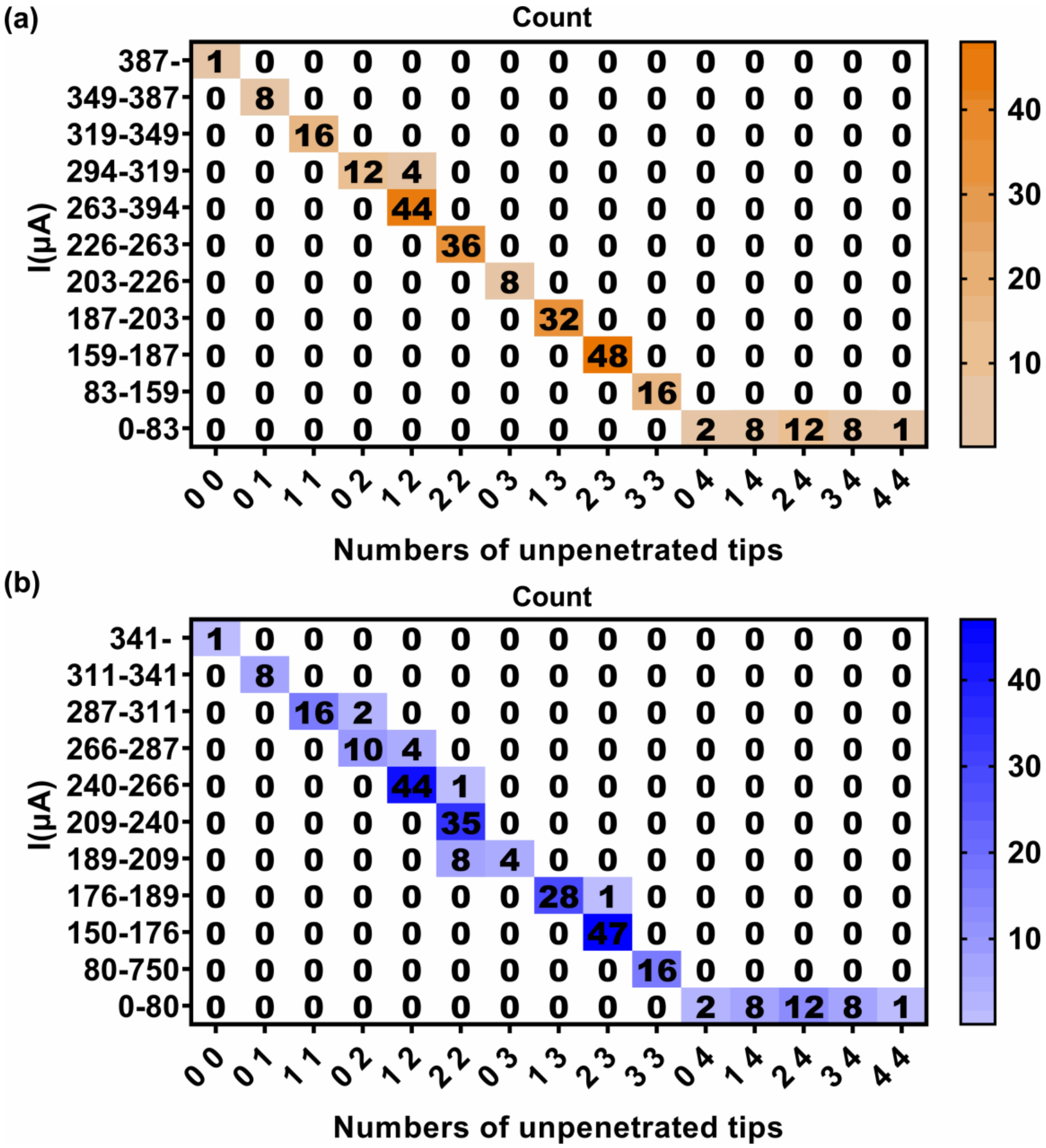

Figure S5. Heat map of the current interval of the transdermal state of (a)A-C and (b)A-D of the 4×4 model.

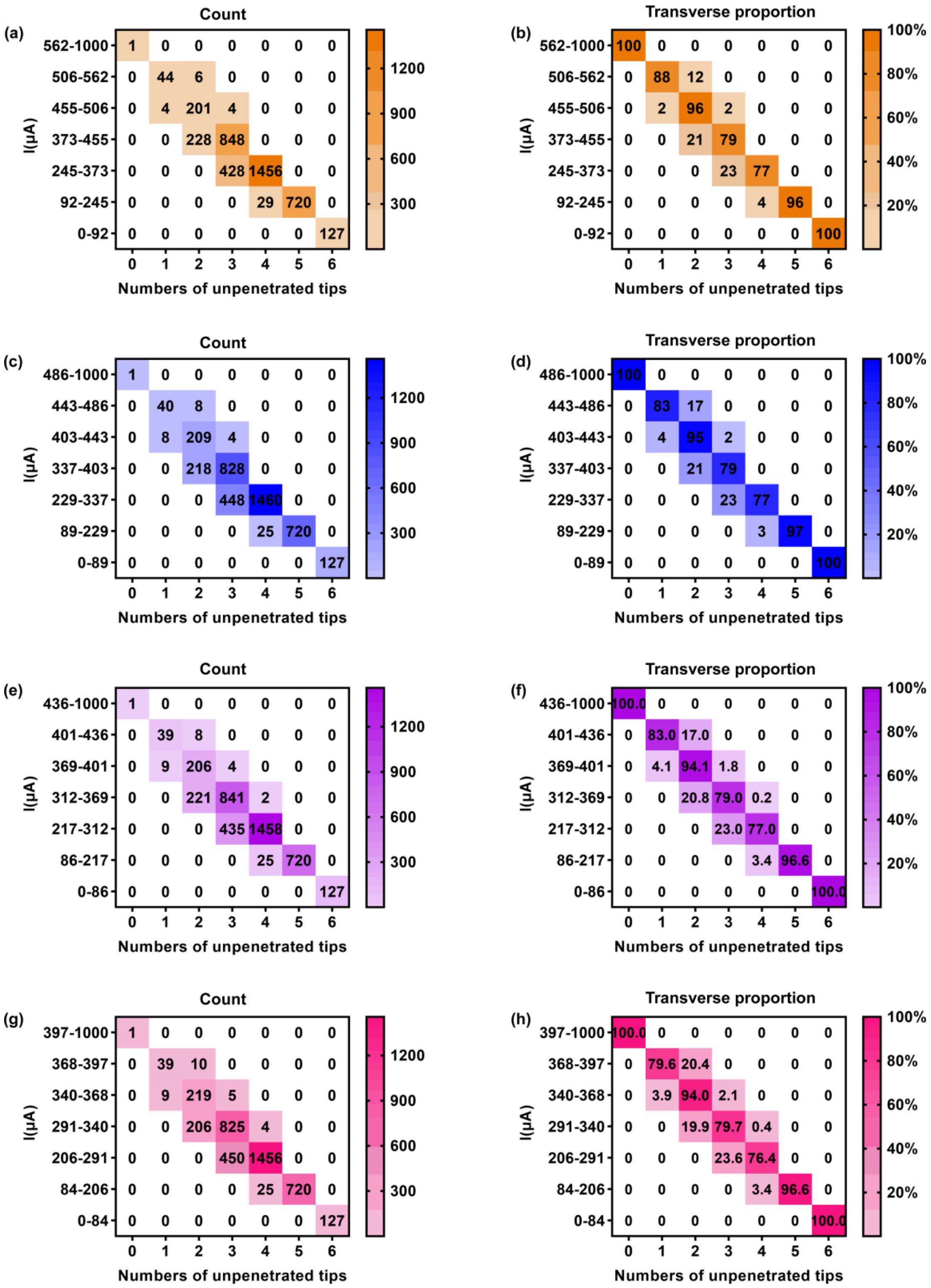

Figure S6. The counting heat map of the current interval of the $N_{max\text{-}s}$ in a single sheet of (a)A-C, (c) A-D, (e) A-E, and (g) A-F of the 6×6 model. The percentage heat map of the current interval of the $N_{max\text{-}s}$ in a single sheet of (b)A-C, (d) A-D, (f) A-E, and (h) A-F of the 6×6 model.

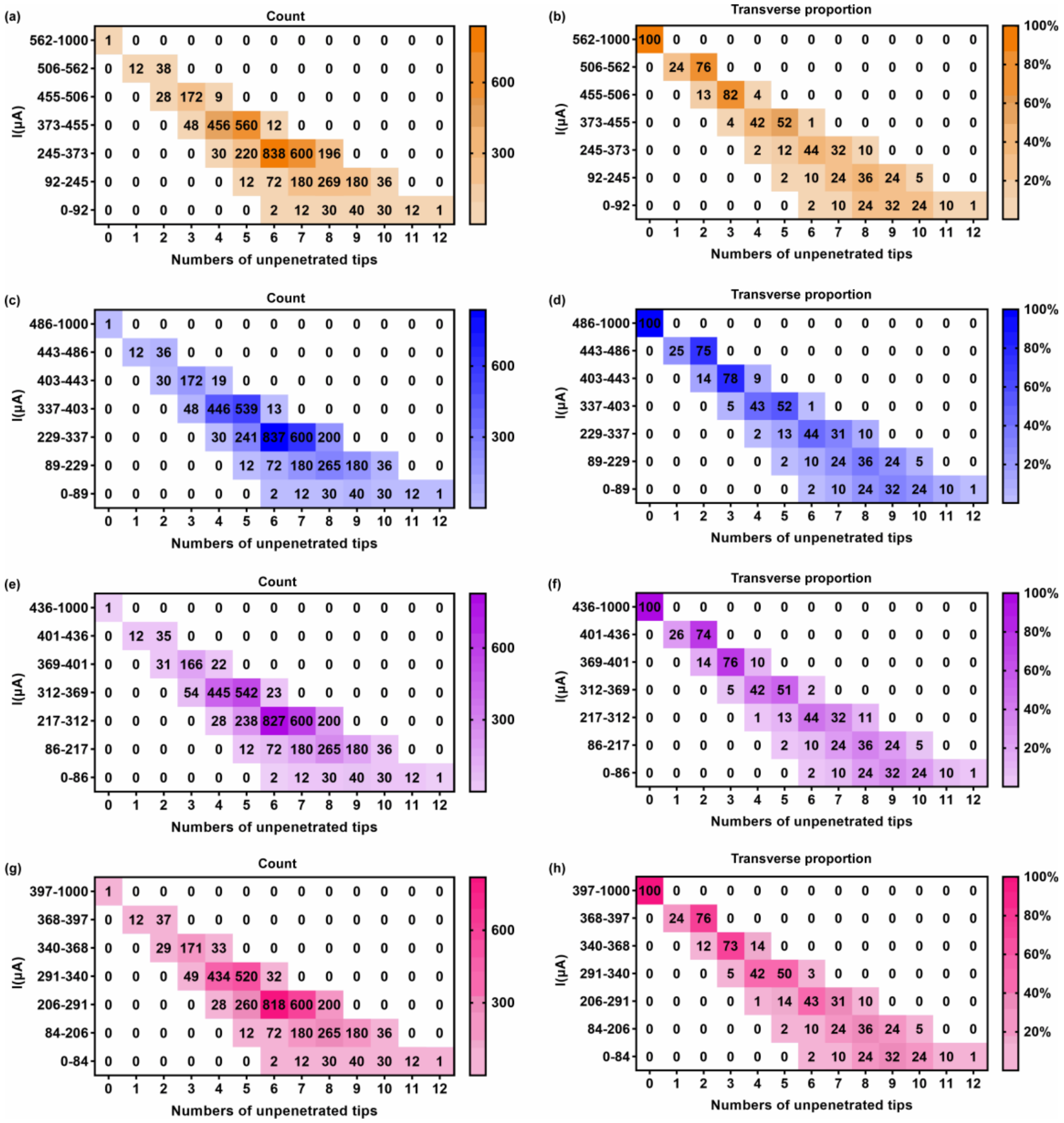

Figure S7. The counting heat map of the current interval of the N_{total} of (a)A-C, (c) A-D, (e) A-E, and (g) A-F of the 6×6 model. The percentage heat map of the current interval of the N_{total} of (b)A-C, (d) A-D, (f) A-E, and (h) A-F of the 6×6 model.
